# Optimal sampling designs for accurate estimation of parasite clearance in the context of artemisinin resistance

**DOI:** 10.1186/1475-2875-11-S1-P39

**Published:** 2012-10-15

**Authors:** Jennifer A Flegg, Philippe J Guerin, Francois Nosten, Arjen M Dondorp, Rick M Fairhurst, Duong Socheat, Steffen Borrmann, Anders Björkman, Andreas Mårtensson, Mayfong Mayxay, Paul Newton, Delia Bethell, Youry Se, Harald Noedl, Abdoulaye A Djimde, Nicholas J White, Kasia Stepniewska

**Affiliations:** 1 Worldwide Antimalarial Resistance Network (WWARN), Asia Regional Centre, Bangkok, Thailand; 2 Centre for Tropical Medicine, Nuffield Department of Clinical Medicine, University of Oxford, Oxford, UK; 3 Mahidol-Oxford Tropical Medicine Research Unit, Mahidol University, Bangkok, Thailand; 4 Shoklo Malaria Research Unit, Mae Sot, Thailand; 5 Laboratory of Malaria and Vector Research, National Institute of Allergy and Infectious Diseases, Bethesda, Maryland USA; 6 Center for Parasitology, Entomology and Malaria Control, Pnom Penh, Cambodia; 7 Kenya Medical Research Institute/Wellcome Trust Research Programme, Kilifi, Kenya; 8 Unit of Infectious Diseases, Karolinska Institutet, Karolinska Hospital, Stockholm, Sweden; 9 WellcomieTrust-Mahosot Hospital-Oxford Tropical Research Collaboration, Mahosot Hospital, Vientiane, Laos; 10 Department of Immunology and Medicine, Armed Forces Research Institute of Medical Sciences (AFRIMS), Bangkok, Thailand; 11 Institute of Specific Prophylaxis and Tropical Medicine, Medical University of Vienna, Vienna, Austria; 12 Malaria Research and Training Centre, University of Bamako, Bamako, Mali

## Background

The emergence of artemisinin resistance in South East Asia threatens the efficacy of artemisinin derivatives (AD). Since the pharmacodynamic hallmark of AD is rapid parasite clearance, the clinical phenotype of slow clearance characterises resistance. This indicator remains critically important to monitor the extent of the problem in the absence of molecular marker(s) associated with artemisinin resistance and lack of sensitivity of current in vitro tests. Frequent parasite counts are needed to define clearance rate but it is uncertain what sampling frequency is required to ensure reliable estimates.

## Materials and methods

WWARN established a study group project to assess this question. Twelve studies with 4552 patients with frequent parasite counts, from Cambodia, Thailand, Laos, Bangladesh, Mali, Tanzania and Kenya were included in the analysis. Patients were treated with artesunate alone or in combination with a partner drug. The WWARN Parasite Clearance Estimator [1,2] was used to produce standardized estimates of parasite half-life (HL). Parasitaemia-time profiles with 6-hourly parasite counts available in the first 48 hours (h) were used to examine the effect of different sampling strategies on HL estimates - four measurement schedules were investigated at: (a) 0,6,12,24 or (b) 0,6,18,24 or (c) 0,12,18,24 or (d) 0,12,24 h and then every 12h. Bootstrapping was used to estimate the sampling distribution of HLs for subsets of the profiles with different distributions of HLs. A simulation study was performed to investigate optimal schemes. Parasite counts were generated from an overdispersed Poisson distribution based on the variability observed in the study data and assuming a first order elimination process.

## Results

The median (range) of estimated HLs was 3.1 h (0.6-17.4). Estimates varied significantly between study location and year (p<0.001), with median HLs ranging from 1.9-6.3 h and the coefficient of variation ranging from 26-52% between studies. Nearly 50% (2251/4552) of the profiles had 6-hourly counts. In these profiles the median (range) for the difference between the original HL estimate and that from the 4 schemes were -0.02 (-3.4 to 3.8), -0.06 (-3.3 to 3.5), -0.09 (-3.6 to 3.4), -0.15 (-5.0 to 3.6) h, respectively. The overestimation of the HL by the restricted schemes was greater for profiles with short reference HL. Boostraping showed that the median HL was overestimated by the 4 schemes in the majority of bootstrap samples. The schemes overestimated the proportion (%) of profiles with a HL >3h, on average by 6,7,9,12% in bootstrap samples with slow clearing parasites (50% of HL longer than 3h) and 39,44, 54, 72% in bootstrap samples with fast clearing parasites (20% of HL longer than 3h), relative to the scheme with 6 hourly measurements. A number of alternative sampling designs derived from the simulation study will be presented and discussed.

## Conclusion

Our data indicate that the estimation of HL is dependent on sampling times for fast clearing parasites. 12 hourly counting is satisfactory in patients with slow clearance but the estimation of short HLs requires more sophisticated sampling schemes. Suggested schemes will need to be tested in a clinical study.
